# Physiological paradigm for assessing reward prediction and extinction using cortical direct current potential responses in rats

**DOI:** 10.1038/s41598-024-59833-7

**Published:** 2024-05-07

**Authors:** Yoshiki Matsuda, Nobuyuki Ozawa, Takiko Shinozaki, Yoshitaka Tatebayashi, Makoto Honda, Toshikazu Shinba

**Affiliations:** 1 Sleep Disorders Project, Department of Psychiatry and Behavioral Sciences, Tokyo Metropolitan Institute of Medical Science, 2-1-6 Kamikitazawa, Setagaya-ku, Tokyo, 156-8605 Japan; 2 Department of Psychiatry, Shizuoka Saiseikai General Hospital, 1-1-1 Oshika, Suruga-ku, Shizuoka, 422-8527 Japan; 3 Research Division, Saiseikai Research Institute of Health Care and Welfare, 21F Mita-Kokusai Building, 1-4-28 Mita, Minato-ku, Tokyo, 108-0073 Japan

**Keywords:** Reward prediction, DC potential, Frontal cortex, Medial forebrain bundle, Anhedonia, Neurophysiology, Reward

## Abstract

Anticipating positive outcomes is a core cognitive function in the process of reward prediction. However, no neurophysiological method objectively assesses reward prediction in basic medical research. In the present study, we established a physiological paradigm using cortical direct current (DC) potential responses in rats to assess reward prediction. This paradigm consisted of five daily 1-h sessions with two tones, wherein the rewarded tone was followed by electrical stimulation of the medial forebrain bundle (MFB) scheduled at 1000 ms later, whereas the unrewarded tone was not. On day 1, both tones induced a negative DC shift immediately after auditory responses, persisting up to MFB stimulation. This negative shift progressively increased and peaked on day 4. Starting from day 3, the negative shift from 600 to 1000 ms was significantly larger following the rewarded tone than that following the unrewarded tone. This negative DC shift was particularly prominent in the frontal cortex, suggesting its crucial role in discriminative reward prediction. During the extinction sessions, the shift diminished significantly on extinction day 1. These findings suggest that cortical DC potential is related to reward prediction and could be a valuable tool for evaluating animal models of depression, providing a testing system for anhedonia.

## Introduction

Reward prediction is a fundamental cognitive process that involves the expectation of positive outcomes, leading to heightened motivation and behavioral adjustment^1–3^. This process is primarily regulated by midbrain dopaminergic activity, with dopamine neurons initially responding to unconditioned stimuli during the early stages of classical conditioning through intracranial self-stimulation (ICSS) at the medial forebrain bundle (MFB), hypothalamus, lateral habenula, and ventral tegmental area. However, after conditioning is complete, the dopamine neurons switch their responsiveness from the reward to conditioned stimuli, such as visual and auditory cues, exhibiting a phasic shift in activity^4^.

Brain activity associated with reward prediction involves the temporal element and changes over time until the reward is obtained. Therefore, identifying brain activity related to prediction necessitates the use of physiological measures with high temporal resolution, such as electroencephalogram (EEG), in addition to frequently used brain imaging techniques^5^. Event-related slow or direct current (DC) potential is an EEG component that arises during movement preparation or predictive anticipation of upcoming rewarding events while awaiting reward completion^6–8^. Although this slow potential can be extracted during alternating current (AC)-EEG recording, accurate capture of the phenomenon becomes challenging owing to the decrease in amplitude with a short time constant^9^. Conventional AC-EEG recording typically incorporates a low-frequency filter set at or above 0.5 Hz, truncating observable changes in cortical and subcortical DC brain activity^10^. Consequently, a DC amplifier with an infinite time constant is preferable for slow or DC potential recording.

Therefore, we focused on brain DC potential and assessed reward prediction. The brain DC potential exhibits fluctuations ranging from seconds to hours in relation to brain activities^11,12^, including arousal levels^12^, and motivation-related behaviors^13^. Previously, DC brain potential responses have been reported in rats when presented with reward cues during conditioning training utilizing ICSS or feeding^6,8^. Based on these findings, we neurophysiologically evaluated reward prediction through DC potential in rats.

This study investigated the role of cortical DC potential in prediction of electrical stimulation of the MFB and evaluated its quantitative change as a physiological indicator of reward prediction.

## Results

### Frontal DC potentials exhibit the strongest negative shift during reward prediction

**Figure 1:**
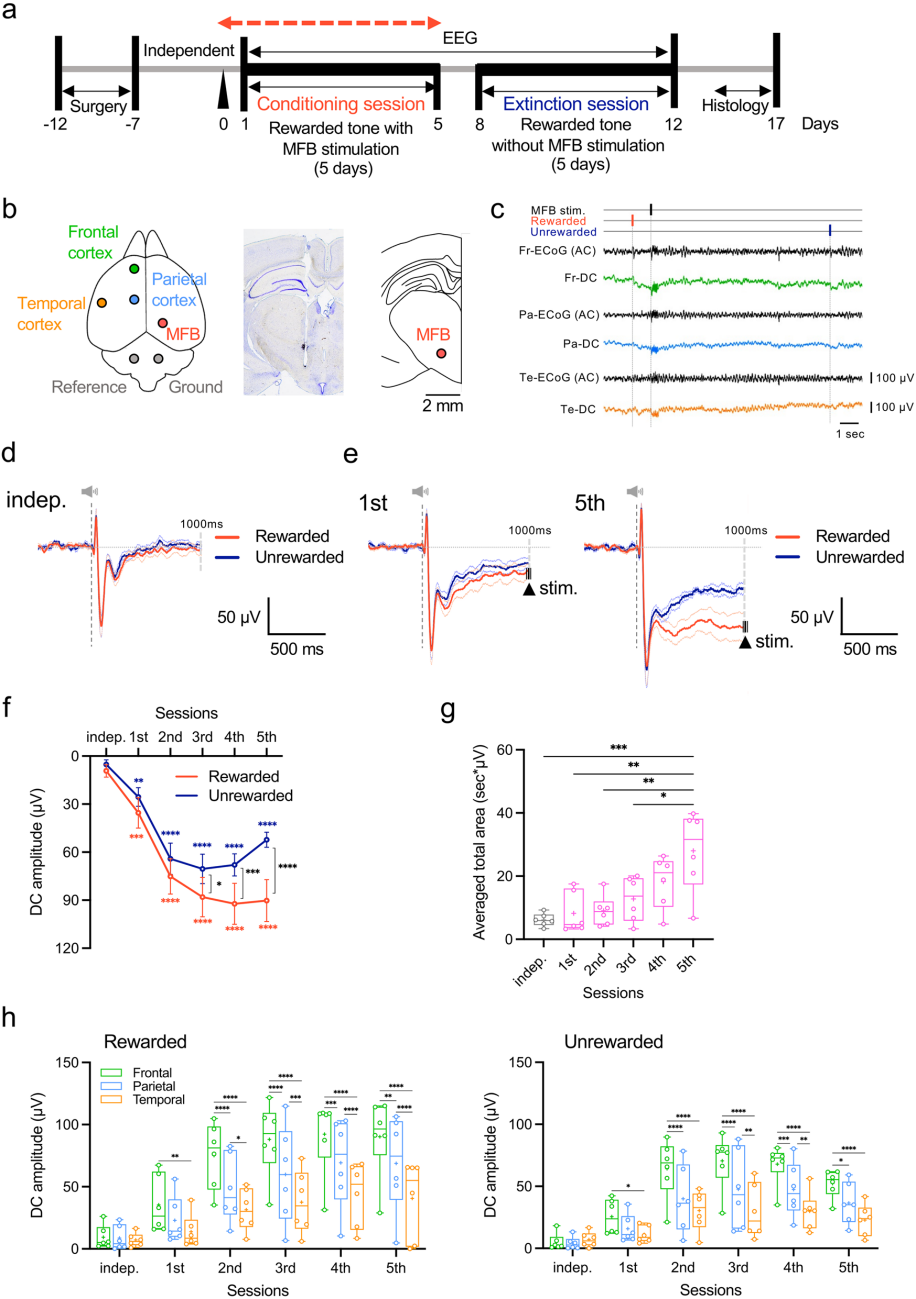
Changes in cortical direct current (DC) potential responses during the progression of two-tone (7 and 11 kHz) discrimination learning using passive electrical stimulation of the medial forebrain bundle (MFB). (**a**) Schematic diagram of a 5-day conditioning session for discriminating between two tones for reward prediction. The sessions involved association learning and extinction through discriminative conditioning using the two tones and electrical stimuli to the MFB. (**b**) Position of non-polarized Ag/AgCl screw electrodes for cortical DC potential recording and insertion of the stimulation electrode into the MFB. (**c**) DC potential and AC-EEG recordings during discriminative conditioning sessions. Negative shifts were observed in the traces of DC recordings upon presentation of the rewarded and unrewarded tones. *Fr* frontal, *Pa* parietal, *Te* temporal. (**d**) Average waveforms of the DC potential response of the frontal cortex to two independent tones (rewarded and unrewarded tones using 7 or 11 kHz) without MFB stimulation. (**e**) Gradual appearance of DC potentials in the frontal cortex after presenting the rewarded and unrewarded tones on days 1 and 5 of the conditioning session. (**f**) Stepwise changes in the mean amplitude of the frontal cortical DC potentials during discriminative conditioning of reward prediction through the presentation of the rewarded and unrewarded tones. (**g**) Chronological changes in the mean integrated area of the difference in DC potential responses elicited by the presentation of both tones during discrimination conditioning sessions. (**h**) Chronological changes in the mean amplitude of DC potentials in the frontal, parietal, and temporal cortices during discriminative conditioning of reward prediction for rewarded. *P < 0.05, **P < 0.01, ***P < 0.001, ****P < 0.0001.

We initially analyzed DC potential responses in the frontal, parietal, and temporal cortices to independent tone presentations without stimulation to the histologically identified MFB (Fig. 1a–c, Supplementary Fig. 1). The average DC potential response in the frontal cortex to two independent tones (7 kHz and 11 kHz) without any concurrent MFB stimulus showed nearly identical waveforms (Fig. 1d). The next day, we started a 5-day conditioning session to associate one of the two tones with the MFB stimulus (Fig. 1a,c). On day 1 of the discriminative conditioning session, negative shifts in the frontal DC potential occurred following the rewarded and unrewarded tones, compared with baseline levels before each tone presentation (Fig. 1e; left). These negative shifts increased in magnitude as the discriminative conditioning progressed (Fig. 1e; right, *F*_(5, 25)_ = 34.81, *P* = 0.0003; Rewarded, *P* = 0.0069; Unrewarded, sample size = 6, actual power = 0.8485; logBF_10_ = 16.988, indicating substantial evidence against the null hypothesis; Fig. 1f). From the 3rd conditioning day, the frontal DC potential response to the rewarded tone exceeded that of the unrewarded tone (Fig. 1f). Furthermore, the integrated area representing the difference in DC potential responses in the frontal cortex between the two tones reached its maximum on day 5, similar to the parietal and temporal cortex (Fig. 1g, Supplementary Fig. 2a,b). The amplitude difference between the two tones in frontal DC potential amplitude during the discriminative conditioning session exhibited a significantly greater increase starting from day 2 of the session, compared with the parietal and temporal cortices, reaching its peak amplitude earlier (*F*_(2, 10)_ = 14.67, *P* = 0.0011, logBF_10_ = 5.483; Rewarded, *F*_(2, 10)_ = 15.06, *P* = 0.0010, logBF_10_ = 3.068; Unrewarded, sample size = 4, actual power = 0.8435; Fig. 1h).

### Frontal cortex plays a critical role in discriminating reward prediction signal

**Figure 2:**
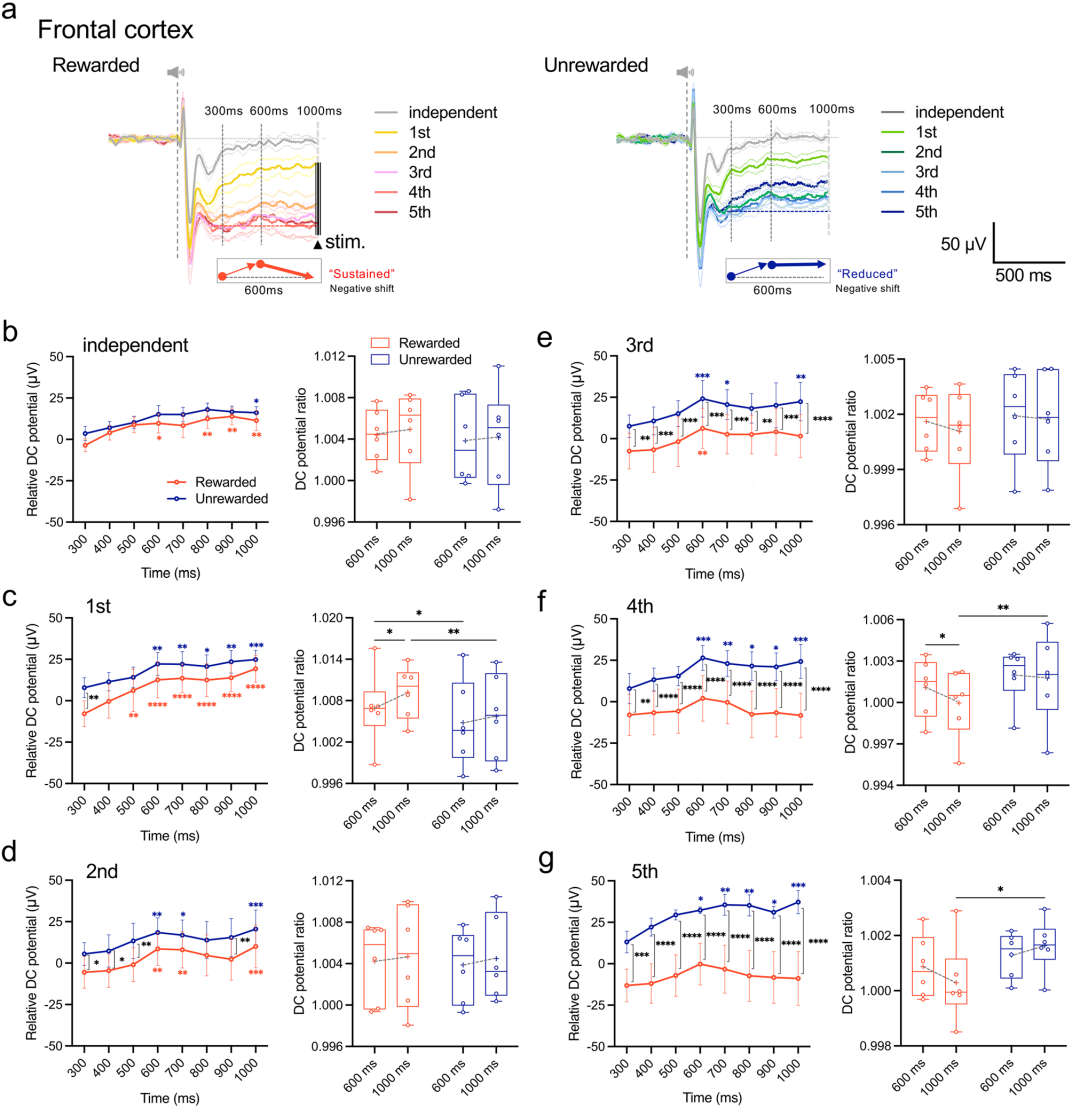
Characteristics of the DC potential response profile related to reward discrimination in the frontal cortex. (**a**) Superimposed averaged waveforms of the daily DC potential response were recorded from the frontal cortex for 1000 ms after the presentation of rewarded (left) and unrewarded (right) tones. Diagram of the DC potential response pattern to the presentation of the discriminative tones on day 4 of the discrimination conditioning. On day 4 of the discrimination conditioning, after the presentation of the rewarded tone, the DC potential increases up to 600 ms and returns to its pre-elevation value toward 1000 ms, showing a “sustained” negative shift, whereas the presentation of the unrewarded tone causes a “reduced” negative shift in the direction of baseline return. (**b**–**g**) Changes in daily relative DC potential values in the time window from 300 to 1000 ms after tone presentation (left), and the ratio of relative potential values at 600 ms and 1000 ms to the relative potential value at 300 ms (right). Tukey’s multiple comparisons test. Relative DC potential was calculated as the difference from the averaged potential at 300 ms for both rewarded and unrewarded tones in 6 rats. *P < 0.05, **P < 0.005, ***P < 0.001, ****P < 0.0001.

Next, we analyzed changes in the DC potential in the frontal, parietal, and temporal cortices during the 300–1000 ms interval by calculating the difference from the averaged amplitude for rewarded and unrewarded tones from 6 rats at 300 ms (relative DC potential, Fig. 2). On day 1 of the discriminative conditioning session, we observed a positive shift in the DC potential amplitude, with statistical significance observed from 600 ms after both rewarded and unrewarded tones (*F*_(7, 35)_ = 6.442, *P* < 0.0001; Rewarded, *P* = 0.0039; Unrewarded, sample size = 5, actual power = 0.8414, logBF_10_ = 61.656; Fig. 2c). On day 3, while maintaining this trend, we detected significantly larger DC potential amplitudes in response to the rewarded tone (*F*_(1, 5)_ = 16.0, *P* = 0.0102, sample size = 5, actual power = 0.8414, logBF_10_ = 58.180; Fig. 2e). By day 4, in addition to this significant difference, we noted a sustained negative shift in the DC potential from 600 ms after the rewarded tone to MFB stimulation (Fig. 2f). In contrast, the DC potential decreased in amplitude from 600 ms following the presentation of the unrewarded tone, indicating distinct response profiles depending on the tone types (*F*_(1, 5)_ = 15.09, *P* = 0.0116, sample size = 5, actual power = 0.8414, logBF_10_ = 57.672; Fig. 2f). Moreover, the ratio of DC potential at 600 ms and 1000 ms to that at 300 ms after tone presentation increased for both tones until day 3 but decreased for the rewarded tone on day 4, whereas it remained unchanged for the unrewarded tone presentation (Fig. 2b–g). Notably, these specific DC potential responses were localized to the frontal cortex and absent in the parietal (Supplementary Fig. 3) or temporal (Supplementary Fig. 4) cortex.

### Chronological changes in auditory evoked potential (AEP) components during discriminative conditioning

**Figure 3:**
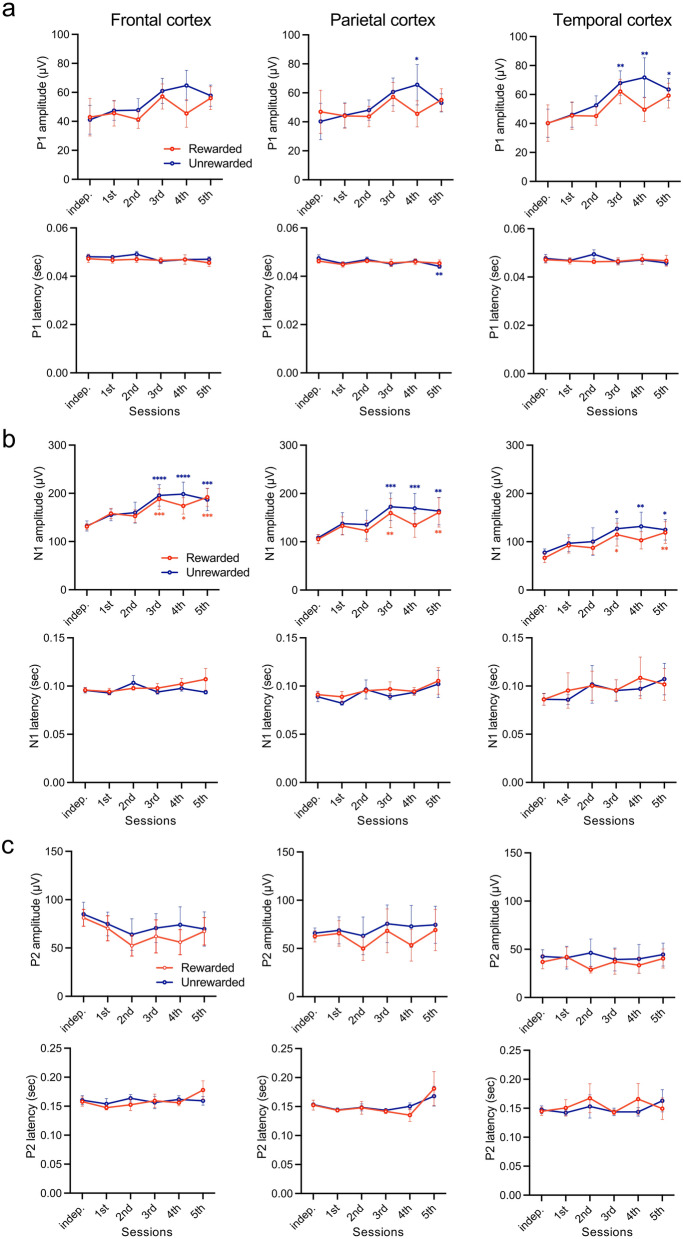
Auditory-evoked potentials (AEPs) responses to rewarded and unrewarded tones in discriminative conditioning. Chronological changes in the mean amplitudes of P1 (B), N1 (C), and P2 (D) at the frontal (left), parietal (center), and temporal (right) cortices during the development of discriminative conditioning (upper; mean amplitude (µV), lower; mean latency (s)). Tukey’s multiple comparisons test of AEPs amplitude and latency in daily sessions for the independent condition. *P < 0.05, **P < 0.01, ***P < 0.001, ****P < 0.0001.

We investigated the temporal changes in AEP components during the discriminative session, distinguishing between rewarded and unrewarded tones (Fig. 3). After day 3, the mean amplitude of the P1 component, elicited by the unrewarded tone presentations, significantly increased in the temporal cortex (*F*_(5, 25)_ = 4.383, *P* = 0.0053, sample size = 6, actual power = 0.8485, logBF_10_ = 11.764; independent: 40.15 ± 9.80 μV vs. day 5: 63.48 ± 7.00 μV; Fig. 3a; top). On day 5, the latency of the P1 component significantly decreased in the parietal cortex compared with the independent condition (*F*_(5, 25)_ = 3.079, *P* = 0.0267, sample size = 6, actual power = 0.8485, logBF_10_ = 8.563; independent: 0.047 ± 0.001 s vs. day 5: 0.044 ± 0.001 s), with no significant difference between the two tones (Fig. 3a; bottom). Furthermore, the mean amplitude of the N1 component (P1–N1) significantly increased after day 3 across the frontal, parietal, and temporal cortices, for both rewarded and unrewarded tone presentations (*F*_(5, 25)_ = 5.594, *P* = 0.0014, sample size = 6, actual power = 0.8485, logBF_10_ = 13.304, independent: 132.25 ± 10.41 μV vs. day 5: 187.05 ± 23.12 μV; frontal cortex, *F*_(5, 25)_ = 4.759, *P* = 0.0034, sample size = 6, actual power = 0.8485, logBF_10_ = 10.536, independent: 108.19 ± 6.65 μV vs. day 5: 163.60 ± 28.58 μV; parietal cortex, *F*_(5, 25)_ = 7.450, *P* = 0.0002, sample size = 6, actual power = 0.8485, logBF_10_ = 11.520, independent: 77.57 ± 7.61 μV vs. day 5: 124.67 ± 21.32 μV; temporal cortex; Fig. 3b; top). However, no significant differences were observed between the two tones (Fig. 3b; top). The latency of the N1 component exhibited similar temporal changes in response to both tones (Fig. 3b; bottom), and there were no significant differences in the amplitude or latency of the P2 component (N1–P2) throughout the conditioning sessions (Fig. 3c).

### Cortical DC potential profile during the extinction of reward prediction conditioning

**Figure 4:**
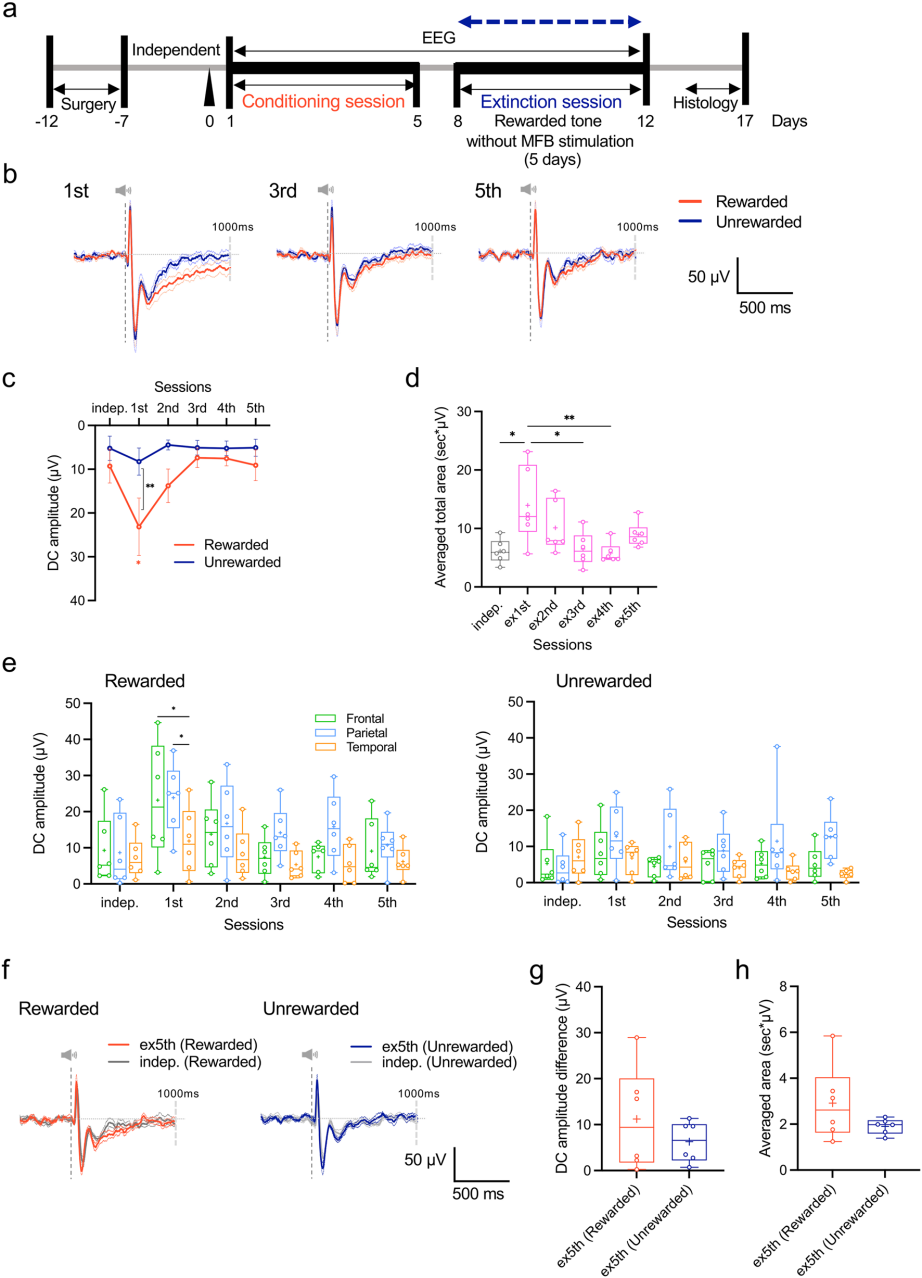
Changes in cortical DC potential responses associated with extinction of discriminative conditioning of reward prediction using two-tone presentations without passive electrical stimulation of the MFB. (**a**) Schematic diagram of a 5-day extinction session for discriminative conditioning of reward prediction using two tones. (**b**) Average waveforms of DC potential responses in the frontal cortex after presentation of rewarded and unrewarded tones on days 1, 3, and 5 of the extinction session. (**c**) Chronological changes in the mean amplitude of the frontal DC potential response to a two-tone presentation during the extinction session. *F*_(5, 25)_ = 2.361, *P* = 0.0693, *P < 0.05, **P < 0.01, two-way (condition × session) ANOVA with Tukey’s multiple comparisons test. (**d**) Chronological changes in the mean integrated area of the differences in DC potential responses elicited by both tone presentations during the extinction sessions. *F*_(5, 30)_ = 4.394, *P* = 0.004, *P < 0.05, **P < 0.01, one-way ANOVA followed Tukey’s multiple comparisons test. (**e**) Comparison of the chronological changes in the mean amplitude of DC potentials in the frontal, parietal, and temporal cortices during the extinction session using the presentation of the rewarded tone. (**f**) Comparison of the waveforms of DC potential responses induced by the presentation of rewarded (left) and unrewarded (right) tones on day 5 of the extinction session with the responses to the corresponding tones in the independent condition before the discriminative conditioning session. (**g**,**h**) Comparison of mean residual amplitudes (**g**) and mean residual integrated areas (**h**) in DC potential response waveforms corresponding to each tone presentation on day 5 of the extinction and independent sessions.

After five conditioning sessions, we initiated an extinction session to disassociate the rewarded tone from the MFB stimulation (Fig. 4a). Initially, frontal DC potentials elicited by the rewarded tone remained significantly higher than those elicited by the unrewarded tones, indicating a persistent negative shift of DC potentials to the rewarded tone after discriminative conditioning (*F*_(1, 5)_ = 9.474, *P* = 0.0275, sample size = 6, actual power = 0.8485, logBF_10_ = 9.870; Fig. 4b,c). However, by day 2 of the extinction session, there was no significant difference in the amplitude of the DC potentials elicited by the rewarded and unrewarded tones (Fig. 4c). Although the amplitude of the frontal DC potential induced by the rewarded tone demonstrated a statistically significant increase compared with the independent baseline DC potential on day 1 of the session, the difference was absent on day 2 and gradually decreased until it returned to the independent DC potential (*F*_(5, 25)_ = 2.720, *P* = 0.0428, sample size = 6, actual power = 0.8485, logBF_10_ = 9.870; Fig. 4c). Conversely, on day 1 of the extinction session, the amplitude of the DC potential induced by the unrewarded tone was slightly higher than that at the baseline level but remained at the baseline amplitude level on day 2 (Fig. 4c; Unrewarded). Additionally, similar to the temporal cortex, a significant decrease occurred in the integral area of the frontal cortex as the extinction session progressed (*F*_(5, 30)_ = 4.394, *P* = 0.0040, sample size = 21, actual power = 0.8187, logBF_10_ = 3.232; Fig. 4d, *F*_(5, 30)_ = 3.204, *P* = 0.0196, total sample size = 21, actual power = 0.8187, logBF_10_ = 1.536; Supplementary Fig. 5b), with no significant change observed in the parietal cortex throughout the session (Supplementary Fig. 5a). The DC amplitude profiles in the parietal cortex were similar to those in the frontal cortex. However, no significant changes in DC potentials were observed in the temporal cortex throughout the extinction session (Fig. 4e). Eventually, the DC potential responses to the rewarded and unrewarded tones in the parietal and temporal cortices returned to the independent baseline level as the extinction session progressed. However, the amplitude was slightly greater in the parietal cortex than in other areas, with delayed reduction in DC potential amplitude (Fig. 4e). To determine the remaining DC potential after reward extinction, the DC potential response waveforms of the rewarded and unrewarded tones on day 5 of the extinction session were compared to those of the corresponding tones during the initial baseline period before the conditioning session (Fig. 4f). However, no significant differences were found in the DC potential amplitude and integrated area (Fig. 4g,h).

### Chronological changes in cortical DC potential response profiles during discriminative extinction

**Figure 5:**
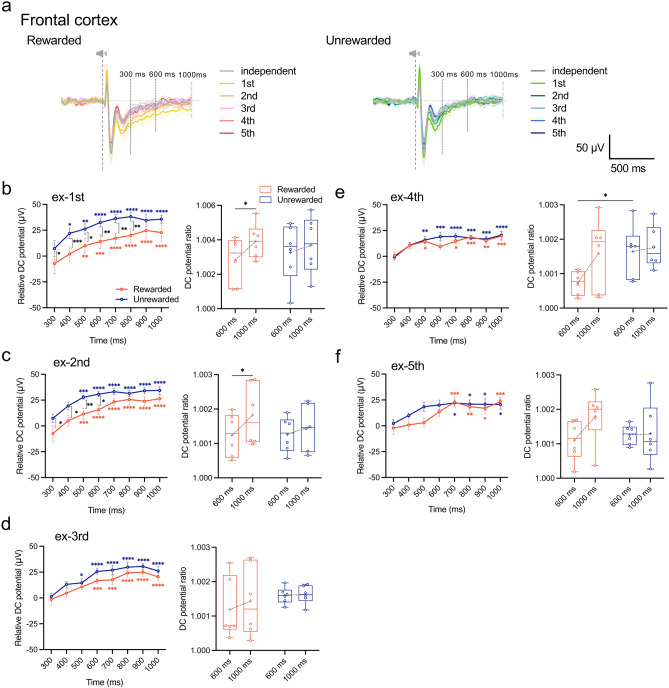
Profiles of the frontal DC potential response to the extinction of discriminative conditioning. (**a**) Superimposed averaged waveforms of the daily frontal DC potential response for 1000 ms after the presentation of rewarded (left) and unrewarded (right) tones during the extinction session. (**b**–**f**) Changes in daily relative DC potential values in the time window from 300 to 1000 ms after tone presentation (left), and the ratio of relative potential values at 600 ms and 1000 ms to the relative potential value at 300 ms (right). Tukey’s multiple comparisons test. *P < 0.05, **P < 0.005, ***P < 0.001, ****P < 0.0001.

We objectively analyzed the DC potential response profiles during the extinction of two-tone discrimination within the time window of 300–1000 ms post-tone presentation, similar to the conditioning sessions (Fig. 5a). On day 1 of the extinction session, the previously observed response patterns during the discernment of the rewarded and unrewarded tones were absent (Fig. 5b). The significant difference in DC potential between the two tones, observed at the start of the extinction session, gradually diminished as the session progressed (Fig. 5b–f; left). Furthermore, the DC potentials response patterns from 300 to 1000 ms post-tone stimulus followed similar temporal trajectories and eventually reached a plateau. The percentage change in the relative DC potentials significantly increased at 1000 ms (vs. 300 ms) in response to the rewarded tone at the beginning of the session (*F*_(1, 5)_ = 8.139, *P* = 0.0357, sample size = 5, actual power = 0.8414, logBF_10_ = 75.644; Fig. 5c; right), but showed no significant difference in the second half of the session. Conversely, the presentation of the unrewarded tone did not lead to a significant percentage change in the relative amplitude of the DC potential throughout the extinction session. In the parietal cortex, the difference in the relative DC potential amplitude resulting from the two-tone presentation disappeared earlier than in the frontal cortex (Supplementary Fig. 6a–e; left). Moreover, on day 1 of the extinction session, the relative DC potential fraction generated by the unrewarded tone decreased significantly by 1000 ms (vs. 300 ms), causing a significant potential shift towards the baseline level (*F*_(1, 5)_ = 22.89, *P* = 0.0050, sample size = 5, actual power = 0.8414, logBF_10_ = 61.543; Supplementary Fig. 6a; right). In the temporal cortex, both stimuli exhibited a consistent DC potential response profile during the extinction session, with no alteration in DC potential proportion (Supplementary Fig. 7).

### Chronological changes in AEPs during discriminative extinction

**Figure 6:**
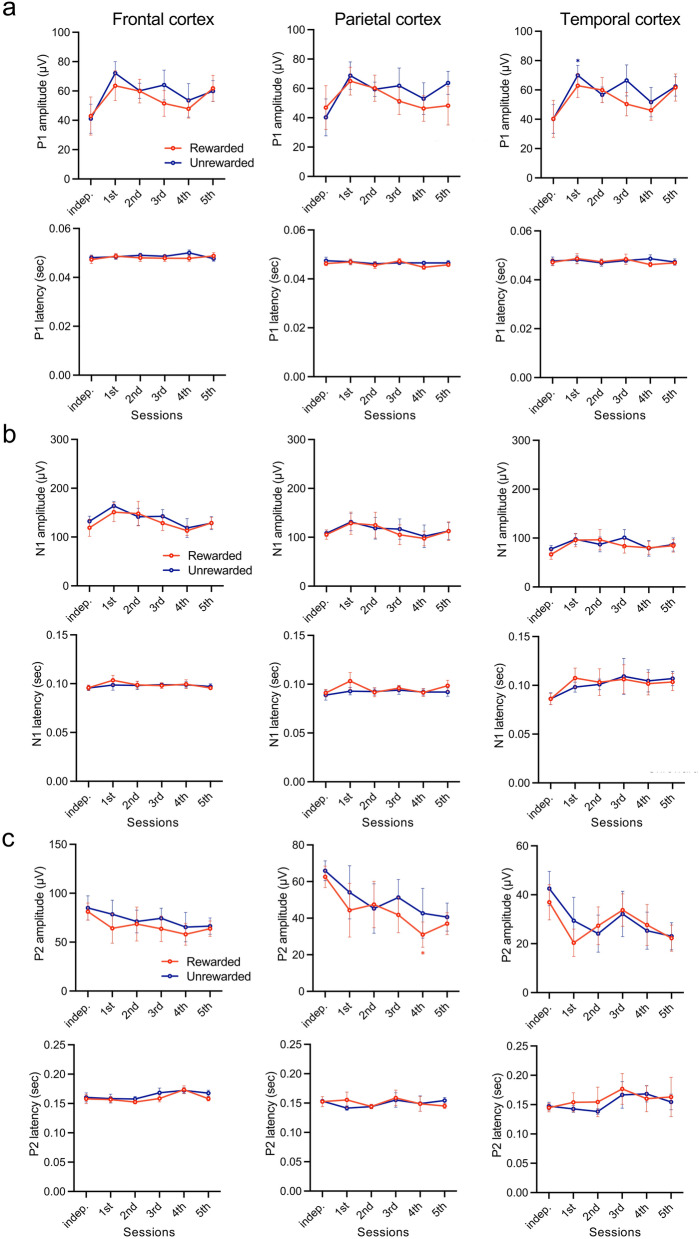
AEPs responses to rewarded and unrewarded tones during the extinction of discriminative conditioning. Chronological changes in the mean amplitudes of P1 (B), N1 (C), and P2 (D) at the frontal (left), parietal (center), and temporal (right) cortices during the extinction of discriminative conditioning (upper; mean amplitude (µV), lower; mean latency (s)). Tukey’s multiple comparisons test of AEPs amplitude and latency during daily extinction sessions for the independent condition. *P < 0.05.

During the extinction of the discriminative conditioning, we analyzed the temporal variations in AEP components (Fig. 6). On day 1 of the extinction session, we observed a significant increase in P1 amplitude in response to the unrewarded tone in the temporal cortex compared to the independent condition (*F*_(5, 25)_ = 2.194, *P* = 0.0337, Tukey’s multiple comparisons test, sample size = 6, actual power = 0.8485, logBF_10_ = 8.580; independent: 40.15 ± 9.80 μV vs. day 1: 70.01 ± 6.80 μV; Fig. 6a; top; temporal cortex). However, on day 2 of the session, no significant changes in P1 amplitude were observed between the extinction and independent condition, and throughout the extinction session, there were no significant differences in the amplitude of the P1 component between the rewarded and unrewarded tone presentations in the temporal cortex (Fig. 6a; temporal cortex). In addition, we observed no significant variations in P1 latency between tone presentations for both tones (Fig. 6a). Furthermore, there were no significant changes in either the amplitude or latency of the N1 (P1–N1) throughout the extinction session (Fig. 6b). However, we observed a significant reduction in P2 (N1–P2) amplitude for rewarded tones in the parietal cortex as the extinction session progressed (independent: 62.60 ± 5.88 μV vs. day 4: 31.04 ± 6.97 μV, Fig. 6c; top; parietal cortex). Similar trends were observed in the frontal and temporal cortices, although there was no significant difference between the responses to the rewarded and unrewarded tones (Fig. 6c; top), and the latency remained unchanged throughout the extinction session (Fig. 6c; bottom).

## Discussion

Here, we present a novel experimental paradigm to investigate reward prediction using cortical DC potential shifts induced through the conditioning of tones with MFB stimulation. After 5-day discriminative conditioning, a significant negative shift in DC potentials was observed in response to the presented rewarded tone compared with those of the unrewarded tone. This shift was predominant in the frontal cortex. Between 600 and 1000 ms after the tone onset, significant differences were observed in the frontal DC potential responses to the tone presentation. Specifically, the rewarded tone elicited a sustained negative shift response, whereas the unrewarded tone demonstrated a reduction in the negative shift. Additionally, discriminative conditioning significantly increased the P1 and N1 amplitudes of the EP with the unrewarded tone, particularly in the temporal cortex. Removing the discriminative conditioning quickly dissipates the negative shift in cortical DC potentials without any remaining difference attributable to the two-tone presentations. The AEP component showed no significant effect on the extinction of reward prediction. These findings have potential implications for utilizing physiological markers of reward prediction, such as anhedonia, in diagnostic and therapeutic approaches for individuals with depression.

Our findings suggest that the negative shift observed in the frontal cortex of the DC potential response related to reward prediction reflects transient information processing, as it almost completely disappeared during extinction. This response may reflect changes in the intensity of psychological motivation, with the frontal cortex playing a crucial role in detecting reward prediction signals. Although initial conditioning showed no distinction in DC potential responses to two-tone presentations, differences in responses related to tone discrimination became more evident as conditioning progressed. The rewarded tone evoked a sustained negative DC shift response, whereas the negative shift response to the unrewarded tone decreased in the frontal cortex, particularly between 600 and 1000 ms after presenting each tone.

In contrast, the acquisition and extinction of reward prediction did not involve AEPs, which are neural responses occurring after the auditory brainstem response and reflect higher-order auditory processing^14^. Different components of the AEP waveform are associated with specific brain regions^15,16^, such as P1, the first positive inflection generated by the auditory thalamus and primary auditory cortex^14^; N1, the first negative deflection associated with the primary auditory cortex; and P2, derived from the associative cortical areas^14^. Given these findings, our results suggest that discrimination acquisition of reward prediction and its extinction do not affect auditory processing in these brain regions. Additionally, a P3-like component, which peaks between 100 and 230 ms after an auditory cue^14^, is related to neural responses in social circuits in various tasks in rats^17–23^ but differs from the peak latency of the cortical DC potentials, suggesting a fundamentally different mechanism.

DC potentials are bioelectrical phenomena generated by cerebral structures. Notably, changes in extracellular ion concentrations, particularly an increase in extracellular K^+^ concentration, can cause depolarization in glial cells, leading to changes in the cortical DC potential^24,25^. These changes have been observed in a heterogeneous manner in layered brain regions such as the cortex and hippocampus^24,26^. Reward prediction occurs topographically in the brain, with the frontal cortex being the most variable area. The polarity of the cortical DC potential shifts is independent of cortical layer structure^24,26^. Our results suggest that the mechanisms generating the DC potential shifts dynamically change during brain activity linked to the acquisition and extinction of reward prediction.

In a groundbreaking study, Pirch et al. investigated slow potentials following warning stimuli using a feeding reward task involving lever pressing and an ICSS task with MFB stimulation under anesthesia^6–8^. Their research illustrated a negative slow potential response in the cortex prior to reward presentation, along with differences in DC potential responses between reinforced and non-reinforced stimuli that align with our findings^6–8^. However, there were differences in experimental conditions between their study and ours, including differences in the duration between the presentation of warning stimuli to reward completion, conditional use of anesthesia, recording of potentials filtered for the AC component of EEG above 3 Hz, lower sampling rates, and fewer potential records for calculating slow potentials^7–9,26,27^. These differences may have limited accuracy in recording slow potential in their study. In contrast, our advanced recording technique enables accurate measurement of unpredictable electrophysiological events by capturing unstable potential phenomena as stable voltage waveforms.

Abnormal reward prediction is closely related to anhedonia, a common manifestation in psychiatric disorders, including depression, characterized by reduced motivation and diminished pleasure^28–30^. Anhedonia reflects disturbances in the hedonic process, encompassing both the anticipatory and consummatory phases of reward processing^29–31^. Motivational anhedonia involves disruptions in the reward prediction process, whereas consummatory anhedonia relates to the loss of pleasure from rewarding stimuli^32–34^. Consummatory anhedonia can be treated with tricyclic antidepressants, whereas motivational anhedonia typically requires monoamine oxidase inhibitors or amphetamines^35^. Other effective treatments for anhedonia may include the dopamine partial agonist aripiprazole and the noradrenaline-dopamine reuptake inhibitor agomelatine^36,37^. These pharmacological studies suggest that the neural mechanisms involved in these processes are different^35^.

To evaluate anhedonia independently, establishing a paradigm that considers its different aspects is essential. Although the sucrose preference test is commonly used, it only measures consummatory anhedonia, representing one aspect of hedonic processing^38,39^. Motivational processes are now considered the core of anhedonia^40^, emphasizing the need to develop a test system capable of identifying this disorder. To achieve this goal, an appropriate animal model that accurately reflects depression pathology should be selected, and an objective method for measuring motivational anhedonia exhibited by the model should be developed.

We developed a novel technique to objectively measure brain activity and indicate the degree of reward prediction. By passively stimulating the MFB and measuring changes in cortical DC potential, we obtained more stable data than those obtained using conventional techniques such as ICSS^6,41,42^ and lever-pressing tasks^41,42^. Our method captured neurophysiological activity preceding positive emotions, which is crucial in assessing motivation for reward prediction and detecting motivational anhedonia in rat hedonic processes. Our social defeat stress (SDS) rat model^43,44^ exhibited high similarity to human depression, rendering it useful in investigating drug responsiveness to antidepressants and sleep disorders^43,44^. Based on the observation that the negative amplitude of DC potentials increases with the acquisition of reward prediction and that there is no residual DC potential upon its elimination, these phenomena can be assessed in the SDS rat model for assessing the physiology of motivational anhedonia. Understanding the relationship between brain DC potentials and depression could lead to more effective treatments for this disorder.

There are some limitations to this study. First, although we identified cortical DC potential change as a brain function related to reward prediction and extinction, the precise mechanisms of this phenomenon remain unclear. Further research is required to identify the direct mechanisms that drive cortical DC potential responses and further investigate the relationship between cortical DC potential generation and deep brain activities, particularly the dopaminergic systems. Second, the possibility that results may vary depending on the condition of electrode position and tone presentation would have to be considered. Because we recorded DC potentials from the cortex contralateral to MFB stimulation due to technical interference issues related to electrode placement, we have to carefully consider potential neurotransmission delays, complexity, and laterality. Unilateral MFB stimulation has been used as a method to induce reward in animals, and EEG recordings from the contralateral side of the stimulating electrode have been used in previous studies^45^. Additionally, the issue of latent inhibition of AEPs due to the random repetition of two tones should be noted. Although equal tone exposure and electrode placement likely minimize habituation, the sample size could still significantly influence the results. Significant differences were found in the analysis of the integrated area, but in some cases, the required sample size was not met. Further data collection will be required in future research to verify this with more certainty. Third, in this study, we did not analyze DC potential responses during reward stimulation. This is because it was difficult to analyze slow electrical phenomena, such as DC potentials, due to many electrical artifacts from the input of the electrical stimulator. Therefore, we could not compare our results with other in vivo recordings. Fourth, to demonstrate the impaired DC potential responses in motivational anhedonia, it is necessary to validate whether the altered reward prediction in depression is indeed reflected in cortical DC potential responses. With this in mind, we plan to apply this testing system to our SDS rat model in future studies^43,44^. This will allow us to investigate both the altered sensitivity of cortical DC response to reward prediction and the presence of residual DC potentials following the extinction of reward prediction. Furthermore, we aim to explore the therapeutic efficacy of pharmacological treatments that could potentially restore these physiological markers. In the present study, a visual examination of the effects of MFB stimulation on behavior in normal rats showed no significant changes, but no quantitative analysis was performed. Quantitative evaluation of the effects of MFB stimulation on behavior in SDS rats remains an important issue. Fifth, we must consider the applicability of this test system to humans since our goal is translational research. However, due to the difficulty of stably recording the DC potentials in humans, this test system utilizing rats may not be directly suitable for humans. Therefore, technical development for stable and accurate recording of DC potentials from the human cortex is required.

Our proposed scoring system holds the potential as a brain marker for depression and stress-related disorders in translational research. Once established, this test system could have several clinical applications. Traditionally, biofeedback therapy for anhedonia is treated with dopamine-related medications and behavioral therapy; however, using physiological indicators, such as EEG and heart rate, as biomarkers could facilitate its development^46^. Although brain DC potentials are similar to the contingent negative variation observed in humans^47^, they have not been used clinically as much as other measures, such as P300^48^. However, our study contributes to clinical research on the relationship between cortical DC potentials and anhedonia. In the future, we anticipate the development of a convenient testing method to objectively evaluate human motivational function by recording EEG from the scalp. Our test system captures the main symptoms of depressive-like behavior in animal models and can serve as a valuable tool for investigating motivational anhedonia.

## Methods

### Animals

This study was approved by the Animal Use and Care Committee of the Tokyo Metropolitan Institute of Medical Science for Ethics of Animal Experimentation. All experimental procedures were performed in accordance with accepted international standards and the guidelines and regulations of the institutional ethics committee. This study was reported in accordance with ARRIVE guidelines. All animals were kept under standard laboratory conditions [12 h light/dark cycle, lights on at 20:00 and lights off at 08:00 (= Zeitgeber time 0; ZT0), room temperature at 20–24 °C], with food and water available ad libitum. A total of 7 male Fisher 334 rats were used for the experiments.

### Electrodes and surgical procedure

Twelve-week-old rats were anesthetized with pentobarbital sodium (60 mg/kg, intramuscular) and secured in a stereotaxic apparatus (SR-6R-HT; Narishige, Tokyo, Japan). The scalp was removed using a surgical scalpel, and a circular craniotomy with a 0.9 mm diameter was performed using a high-speed dental drill. Non-polarized Ag/AgCl screw EEG electrodes (Unique Medical, Tokyo, Japan) with a 1 mm diameter were surgically placed epidurally on the left side of the brain. The electrode position on the skull was determined using the bregma as the reference; the frontal cortex (2.5 mm anterior, 1.5 mm lateral), parietal cortex (2.0 mm posterior, 1.5 mm lateral) and temporal cortex (4.5 mm posterior, 5.5 mm lateral). Additionally, reference and ground electrodes were positioned above the right and left cerebellum (12.5 mm posterior, 1.5 mm lateral). Bipolar stimulating electrodes, consisting of pairs of stainless-steel insulated wires with 1 mm-spaced tip, were stereotaxically implanted in the right MFB (4.5 mm posterior, 1.5 mm lateral, 8.0 mm below the cortical surface to the tip of the deeper electrode) The lead wires from all electrodes were connected to a small socket, and the wires and electrodes were secured on the skull using acrylic resin cement. Antibiotics were administered for three post-surgical days. After a recovery period of over 7 days, the experiment was commenced. The individually housed rats exhibited no signs of discomfort or pain throughout the experiment.

### Cortical DC potential recording

The experiment was conducted during the dark cycle between 10:00 and 15:00 when the rat was awake. To record DC brain potentials, the rat was transferred to a cylindrical experimental cage with a 35 cm diameter located inside a soundproof box in a dark experimental room. From the socket on the skull to which electrodes were connected, DC potential signals were fed through a customized recording cable to a built-in operational amplifier (TL074; Texas Instruments, Dallas, TX, USA) to lower cable impedance and minimize electrical and movement artifacts^12,43,44^. Then, the signals were amplified using DC amplifiers (with a gain of 100 and a high cut filter of 300 Hz; ER-1, Cygnus, Delaware Water Gap, PA, USA), digitized at a sampling rate of 500 Hz, and saved with the Spike2 data acquisition software (Cambridge Electronic Design, Cambridge, UK) after going through an AD converter (Power 1401; Cambridge Electronic Design, Cambridge, UK). At the start of recording, DC potentials between the cortical surface and the cerebellum were set to zero.

### Pre-discriminative conditioning

Prior to five daily discriminative conditioning sessions, baseline responses of DC potentials to auditory stimuli were recorded. During the 1-h recordings, tones of 7 kHz or 11 kHz with 85 dBSPL and 100 ms duration (with the rise and fall being 5 ms) and electrical stimulations to the MFB, traditionally used as the region to activate the brain reward system, were presented independently. Each tone was presented randomly with an equal ratio and at a mean interval of 12 s, ranging from 10 to 14 s. These two-tone auditory signals were output from a speaker installed in a soundproof box via a signal processor (RM1, Tucker-Davis Technologies (TDT), USA) using a real-time processor visual design studio (RPvds, TDT, USA). Electrical stimulations (100 μA, 10 ms duration, 20 times with the inter-stimulus interval of 20 ms) to the MFB were presented via an isolator (SS-104J, Nihon Kohden, Japan) connected to a stimulator (SEN-8203, Nihon Kohden, Japan). The two tones and electrical stimuli were delivered 150 times using Spike2 software (Cambridge Electronic Design, Cambridge, UK).

### Discriminative conditioning session

From the day following the pre-conditioning session, a 1-h discriminative conditioning session was conducted daily for 5 consecutive days. The parameters for the two tones and electrical stimulation to the MFB remained consistent with those used in the pre-discriminative conditioning session. The 7 kHz and 11 kHz tones, each presented 150 times, were randomly assigned as rewarded and unrewarded tones for each rat. Rewarded and unrewarded tones were presented randomly with an equal ratio and at a mean interval of 12 s. Electrical stimulation was applied to the MFB 1000 ms after the presentation of the rewarded tone. The other tone was not followed by stimulation (unrewarded). DC potential was recorded during these procedures.

### Extinction of discriminative conditioning

After the 5-day reward conditioning sessions were completed, extinction sessions were conducted once daily for 5 consecutive days. In the extinction session, rewarded and unrewarded tones were randomly presented with an equal ratio and at a mean interval of 4 s, with the target tone unaccompanied by MFB electrical stimulation.

### Analyses of AEP and DC potential

DC potential signals were averaged 150 times per session from 500 ms before to 1000 ms after the tone presentation to generate ERPs for both rewarded and unrewarded tones. Any DC potential signals with marked movement noise and artifacts were excluded from the analysis. The baseline for the averaged DC potential was defined as the data during the 500 ms period immediately preceding the presentation of tones, and DC amplitude were calculated by subtracting the baseline data from the observed data. Additionally, the integrated area data was calculated as the absolute sum of trapezoidal area differences between DC potentials every 1 ms from 300 to 999 ms after both rewarded and unrewarded tone presentations. Relative DC potential amplitude during the period from 300 to 1000 ms was calculated as the difference from the averaged potential at 300 ms for both rewarded and unrewarded tones in 6 rats, to exclude the effects of amplitude fluctuation caused by the inter-subject differences in AEP profiles. The relative DC potentials were measured as the differences between this average at 300 ms and the DC potential values at 300, 400, 500, 600, 700, 800, 900, and 1000 ms from the tone presentation (Fig. 2).

For AEP analysis, P1, N1, and P2 components were identified based on previous reports^49^. The P1 amplitude was measured as the difference between the observed data and baseline, whereas the N1 and P2 amplitudes were calculated as the differences between the N1 and P2 peaks, respectively. The latencies of AEP components were measured from the onset of the tone.

### Statistical analyses

Statistical analyses were conducted using GraphPad Prism 10 software (La Jolla, CA, USA). The averaged values are presented as mean ± SEM. For statistical significance, the α level was set at 0.05. Two-way repeated analysis of variance (ANOVA) was performed to evaluate differences in the DC amplitude and ratio, as well as AEP amplitude and latency. In these analyses, “session,” “condition,” “area,” and “time” were treated as within-group factors. “Session” indicates the factor for daily conditioning and extinction. “Condition” represents rewarded and nonrewarded factors, “area” signifies the DC potential areas during the period between 300 to 600 ms and that during the 600 to 1000 ms, and “time” denotes the 100 ms interval analysis. Each two-way repeated ANOVA was performed with a pair of these factors (session × condition, session × area, time × condition), followed by post hoc Tukey’s multiple comparisons. For “area,” we also performed one-way ANOVA to compare the data between recording sites of the brain. To capture individual variation within the dataset and to provide a more detailed insight into the dataset, raw data were used for all ANOVA analyses. Whenever statistical significance was found, we estimated the total sample size and actual power using G*power software (ver 3.1.9.6, Franz Faul, Universität Kiel, Kiel, Germany). The parameters utilized for the a priori power analysis for ANOVA with G*Power included an effect size of 0.3333 (partial η^2^ of 0.1), α error probability of 0.05, power (1 − α error probability) of 0.8, correlation among repeated measures of 0.5, and nonsphericity correction ε of 1. The minimum necessary sample size for the present experiment was estimated based on previous research^25,45^. To strengthen the interpretability of our research results, Bayesian repeated measures ANOVAs were conducted using JASP (version 0.18.3, https://jasp-stats.org/). These analyses were designed to generate log Bayes factors (logBF_10_) for the models of interest, specifically “Repeated Measures Factors Models (session × condition, session × area, time × condition),” relative to a null model that included both subject and random slopes. In addition, Bayesian ANOVAs were performed to generate logBF_10_ for the “session” model of interest, relative to a null model that included ID. Based on a previous report^50^, our study adopted the following criteria for determining the strength of evidence based on the logBF_10_ value ranging from anecdotal (0–1.1), to substantial (1.1–2.3), strong (2.3–3.4), and decisive (> 4.6). The data were initially collected from seven rats. Prior to analysis, a quality assurance process was conducted to curate the data. During this process, data from one rat was excluded from the final analysis due to significant potential fluctuations, which were presumably attributed to variations in electrode resistance at the cortical surface.

### Histology

After the experimental sessions were completed, the tip of the MFB electrode was marked by a passing current (− 50 μA, 10 s). The rats were anesthetized with an overdose of pentobarbital sodium (60 mg/kg, intramuscular) and transcardially perfused with 0.01 M PBS (pH 7.4) and 4% paraformaldehyde in 0.01 M PBS with sucrose. The brains were post-fixed in 4% paraformaldehyde overnight and coronally sectioned at a thickness of 40 μm using a cryostat. The resulting serial slices were mounted on glass slides and processed for cresyl violet staining by rinsing with water, ethanol, and xylene, counterstaining with cresyl violet, and coverslipping with a mounting agent. The positions of all electrodes were confirmed by identifying dents on the neocortical superficial layer or tip position and tracks of the MFB stimulating electrode in the histologic tissue, and data were excluded if the electrode position was outside the target brain region. Images of the cresyl violet-stained tissue were captured using a stereomicroscope.

### Supplementary Information

Supplementary Figures.

## Data Availability

The data used to support the findings of this paper are available from the corresponding author upon reasonable request.
