# Supplementary material for: Physiological paradigm for assessing reward prediction and extinction using cortical direct current potential responses in rats
Source: Sci Rep. 2024 May 7;14:10422. doi: 10.1038/s41598-024-59833-7 (PMC11074288; doi:10.1038/s41598-024-59833-7)
Supplement: Supplementary file 1 — Supplementary Figures. [file 41598_2024_59833_MOESM1_ESM.pdf]

Supplementary Materials for

Physiological paradigm for assessing reward prediction and extinction using cortical direct current potential responses in rats

Yoshiki Matsuda, Ph.D., Nobuyuki Ozawa, Ph.D., Takiko Shinozaki, B.D., Yoshitaka Tatebayashi, M.D., Ph.D., Makoto Honda, M.D., Ph.D., Toshikazu Shinba, M.D., Ph.D.

Correspondence to: Yoshiki Matsuda, Ph.D. (matsuda-ys@igakuken.or.jp)

This file includes:

Supplementary Figures 1 to 7

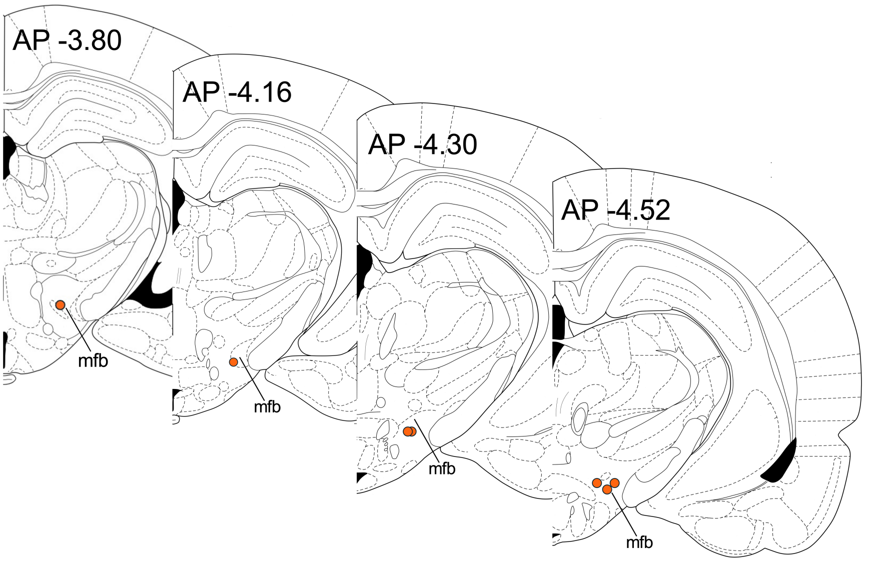

Supplementary Figure 1

Histological localization of the stimulation electrode tips. All electrodes were placed within the medial forebrain bundle (MFB) at the level of the lateral hypothalamus. Each red circle indicates the histologically identified tip position of each MFB stimulation electrode. All electrode tips were located in the ventral aspect of the MFB at the level of the lateral hypothalamus. The number above each section indicates the distance (mm) posterior (-) to bregma. Drawings are adapted from Paxinos and Watson (1986).

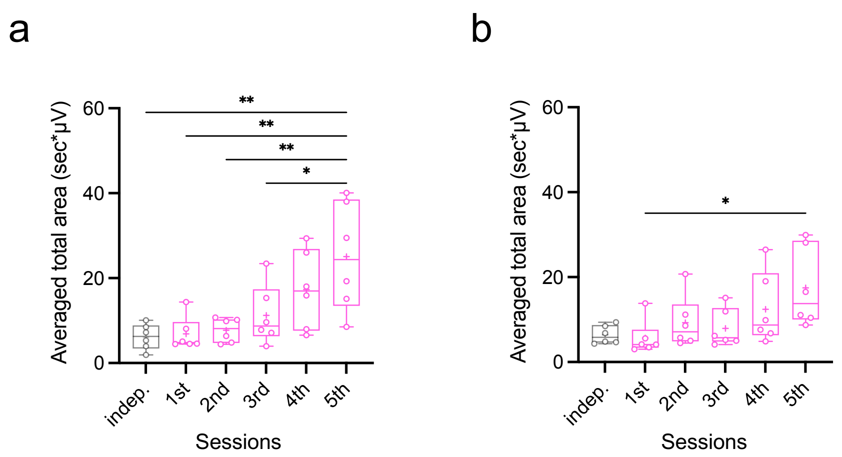

Supplementary Figure 2

Chronological changes during discriminative conditioning in parietal (a) and temporal cortices (b), showing the mean integrated area of the difference between the DC potential waveforms evoked by the two tones. * $P < 0.05$, ** $P < 0.01$, Tukey's multiple comparisons test.

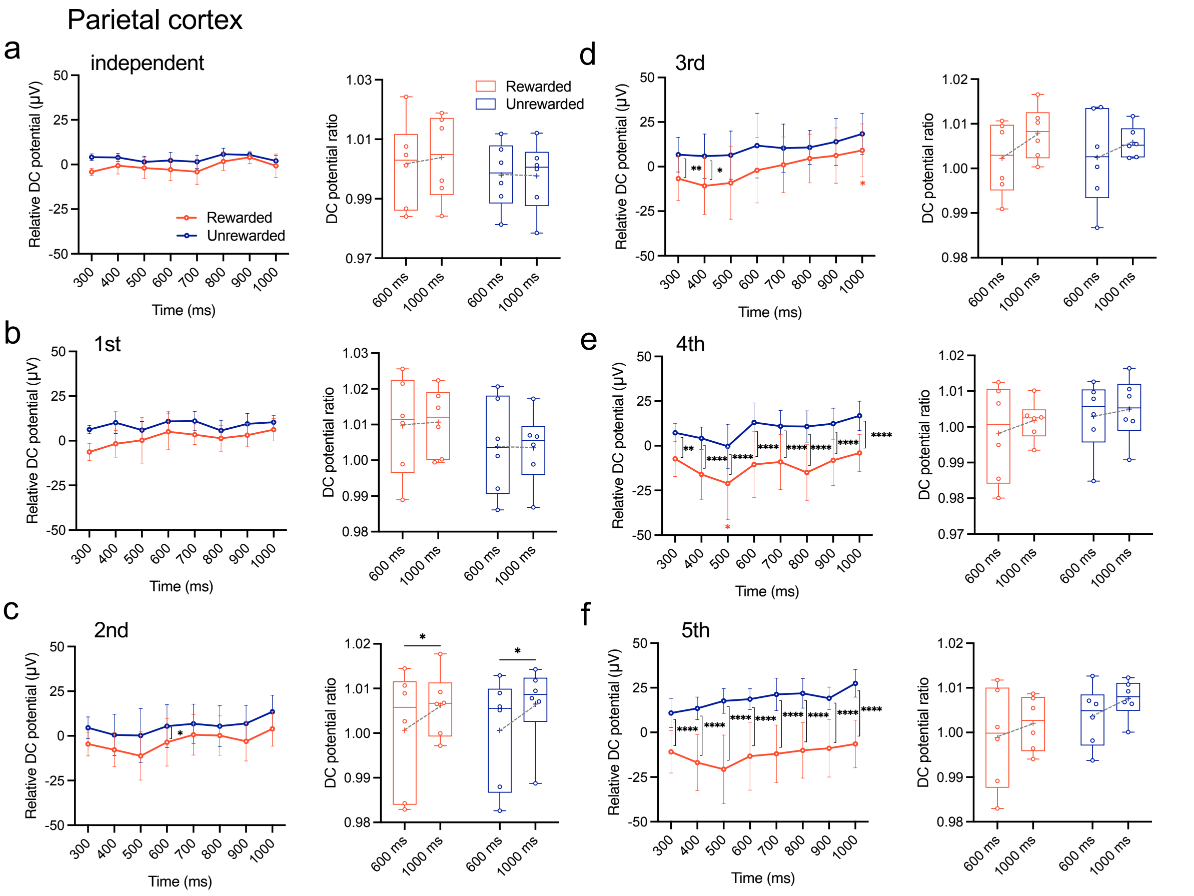

Supplementary Figure 3

DC potential response to discrimination conditioning in the parietal cortex. (**a**–**f**) Changes in daily relative DC potential values between 300 ms and 1000 ms after tone presentation (left), and the ratio of relative potential values at 600 ms and 1000 ms to the relative potential value at 300 ms (right). * $P < 0.05$, ** $P < 0.005$, **** $P < 0.0001$, Tukey's multiple comparisons test.

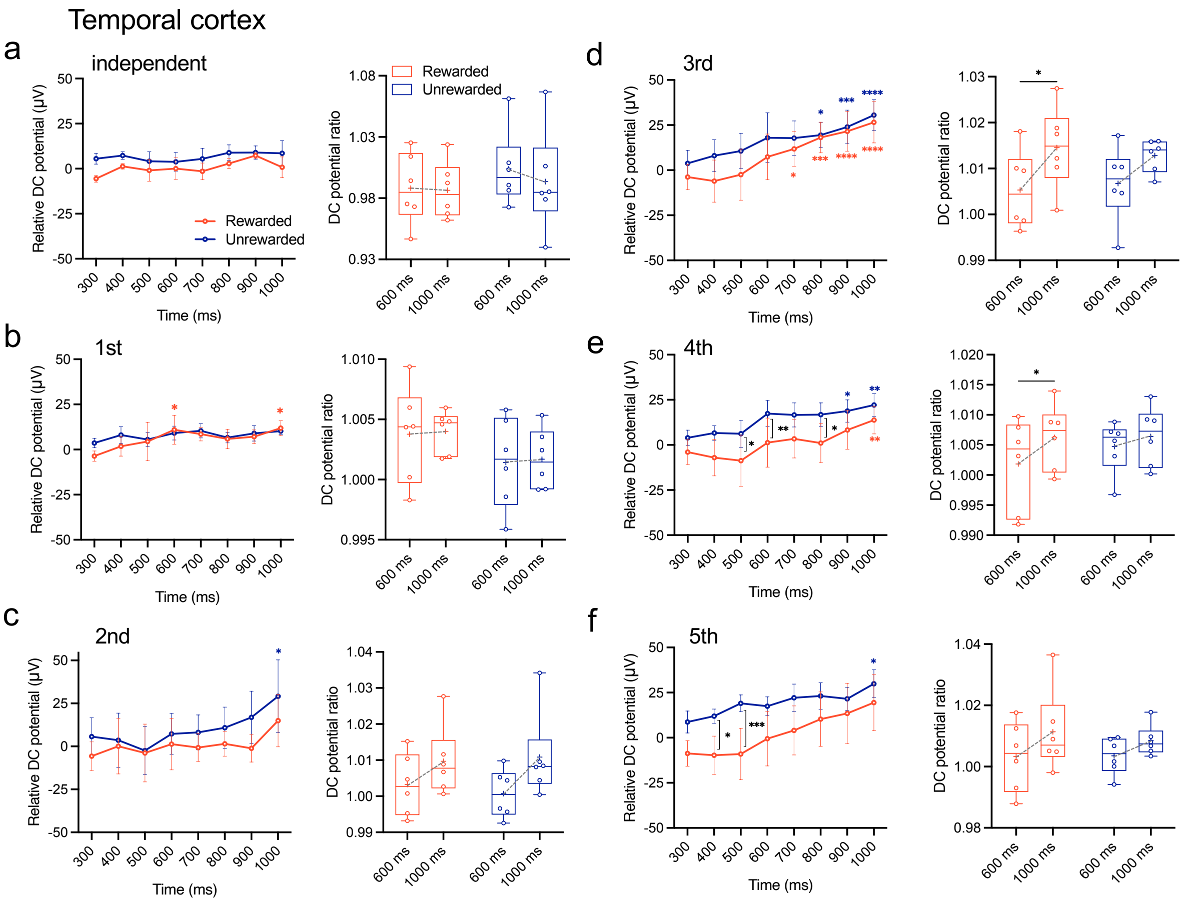

Supplementary Figure 4

DC potential response to discrimination conditioning in the temporal cortex. (**a–f**) Changes in daily relative DC potential values between 300 ms and 1000 ms after tone presentation (left), and the ratio of relative potential values at 600 ms and 1000 ms to the relative potential value at 300 ms (right). * $P < 0.05$, ** $P < 0.005$, *** $P < 0.001$, **** $P < 0.0001$, Tukey's multiple comparisons test.

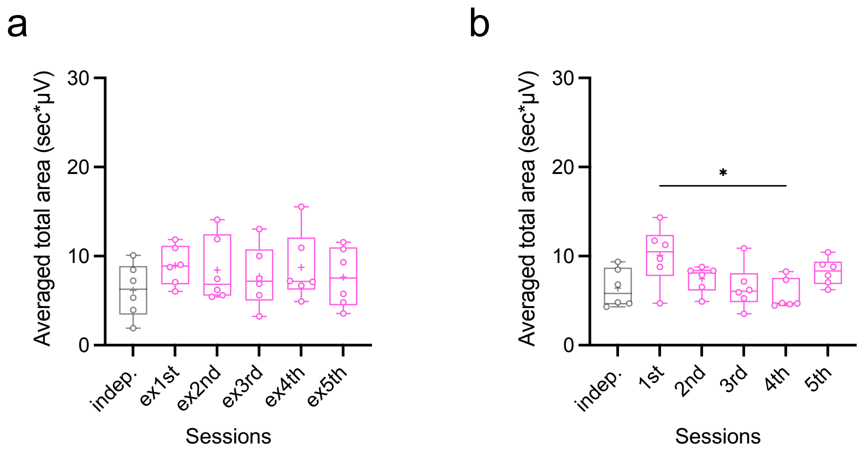

Supplementary Figure 5

Chronological changes in the mean integrated area of DC potentials in the parietal (**a**) and temporal (**b**) cortices during the extinction session of discriminative conditioning. * $P < 0.05$, Tukey's multiple comparisons test.

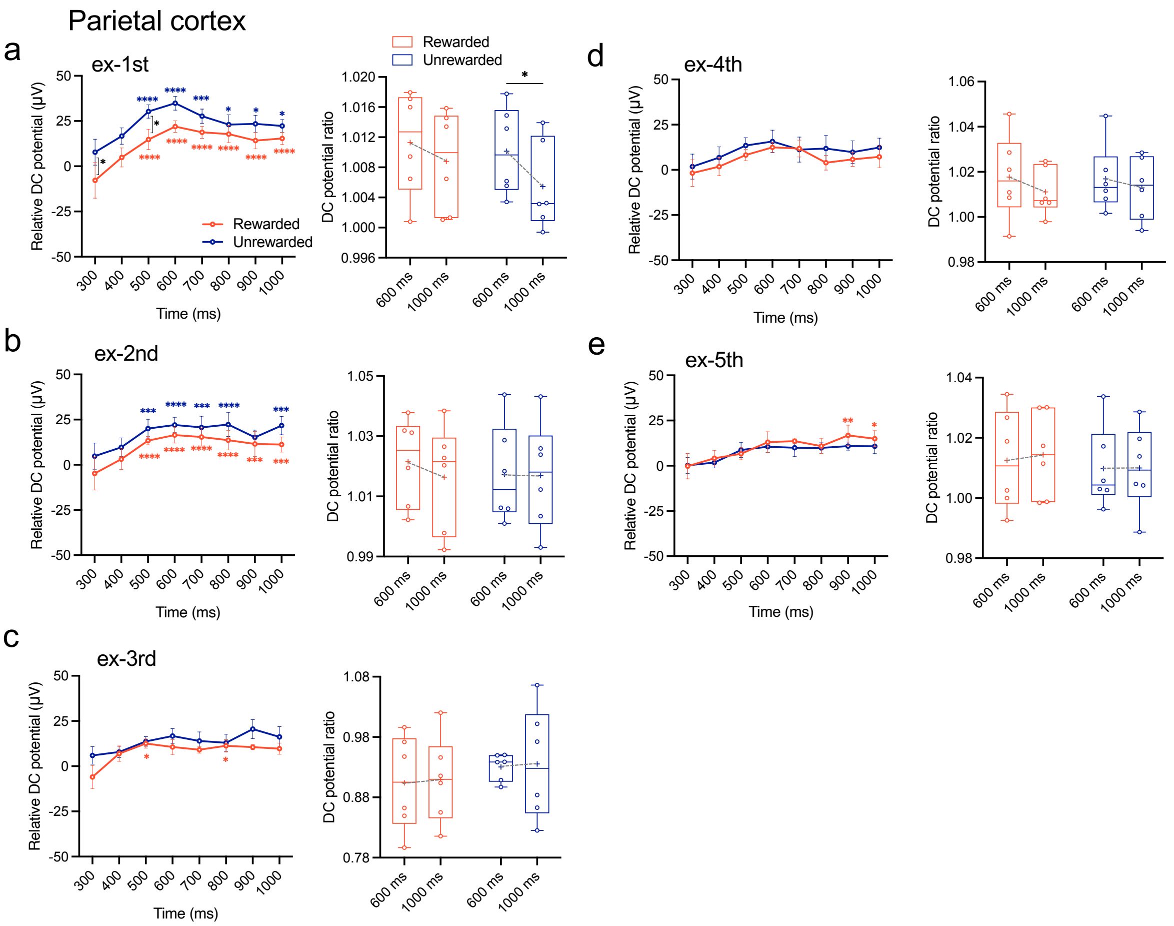

Supplementary Figure 6

DC potential response to the extinction of discrimination conditioning in the parietal cortex. (**a–e**) Daily changes in the relative DC potential between 300 ms and 1000 ms after tone presentation (left), and the ratio of the relative potential at 600 ms and 1000 ms to the relative potential at 300 ms (right). * $P < 0.05$, ** $P < 0.005$, *** $P < 0.001$, **** $P < 0.0001$, Tukey's multiple comparisons test.

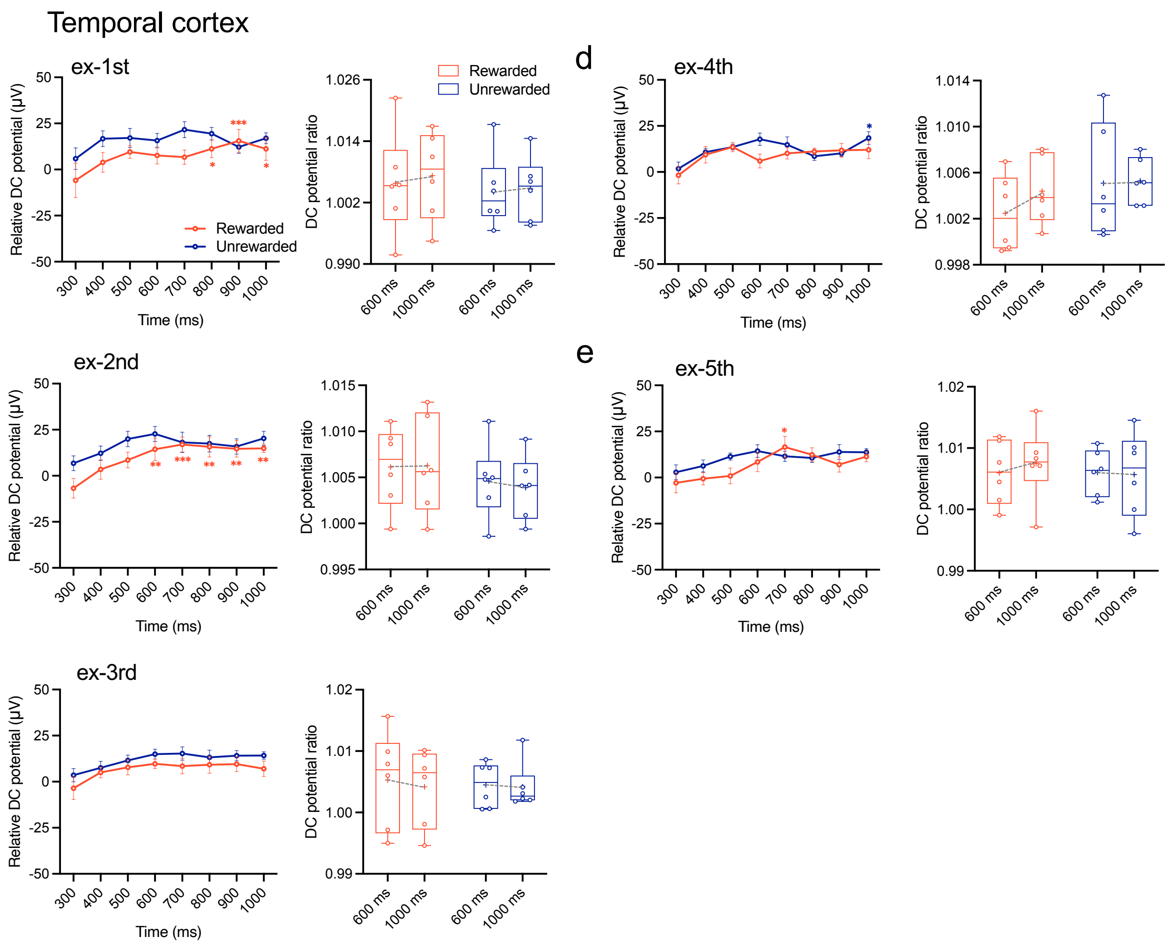

Supplementary Figure 7

DC potential responses to the extinction of discrimination conditioning in the temporal cortex. (**a**–**e**) Daily changes in the relative DC potential at 30–1000 ms after tone presentation (left) and the ratio of the relative potential at 600 ms and 1000 ms to the relative potential at 300 ms (right). * $P < 0.05$, ** $P < 0.005$, *** $P < 0.001$, Tukey's multiple comparisons test.
